# Efficacy of salvage surgery versus re-irradiation for isolated regional lymph node recurrence in patients with nasopharyngeal carcinoma

**DOI:** 10.1186/s12885-024-12259-w

**Published:** 2024-04-16

**Authors:** Yuebing Chen, Yiping Huang, Xiaoqiang Chen, Zhiwei Chen, Xiane Peng, Shaojun Lin, Cheng Lin, Jingfeng Zong

**Affiliations:** 1 Department of Radiation Oncology, Clinical Oncology School of Fujian Medical University, Fujian Cancer Hospital, Fuzhou, 350014 China; 2 Department of Otolaryngology, Fujian Medical University Union Hospital, Fuzhou, Fujian China; 3 Department of Preventive Medicine, School of Public Health, Fujian Medical University, Fuzhou, Fujian China; 4 Fuzhou Center for Disease Control and Prevention, Fuzhou, Fujian China; 5 Key Laboratory of Ministry of Education for Gastrointestinal Cancer, Fujian Medical University, Fuzhou, Fujian China; 6 Fujian Key Laboratory of Translational Cancer Medicine, Fuzhou, Fujian China

**Keywords:** Nasopharyngeal carcinoma, Regional lymph nodes, Recurrence, Re-irradiation, Lymph node dissection

## Abstract

**Background:**

To compare the clinical characteristics and prognoses of patients with isolated regional lymph node recurrent nasopharyngeal carcinoma (irrNPC) who underwent surgery or re-irradiation treatment.

**Methods:**

We retrospectively reviewed 124 irrNPC patients who underwent initial radiotherapy between January 2010 and December 2020. The staging of regional lymph node recurrence was as follows: 75.8% for rN1, 14.5% for rN2, and 9.7% for rN3. Fifty-five patients underwent regional lymph node surgery (Surgery group), and sixty-nine patients received salvage radiotherapy with or without chemotherapy (Re-irradiation group). The survival rate was compared using Kaplan‒Meier analysis and evaluated by the log-rank test. Cox proportional hazard models were used to analyze prognostic factors.

**Results:**

The median follow-up time was 70 months, the 5-year overall survival (OS) was 74%, and the median survival time was 60.8 months. There were no significant differences in 5-year OS (75.6% vs. 72.4%,* P* = 0.973), regional recurrence-free survival (RRFS, 62.7% vs. 71.1%, *P* = 0.330) or distant metastasis-free survival (DMFS, 4.2% vs.78.7%, *P* = 0.677) between the Surgery group and Re-irradiation group. Multivariate analysis revealed age at recurrence, radiologic extra-nodal extension (rENE) status, and recurrent lymph node (rN) classification as independent prognostic factors for OS. The rENE status was an independent prognostic factor for DMFS. Subgroup analysis of the Surgery group revealed that the rN3 classification was an adverse prognostic factor for OS. Age at recurrence ≥ 50 years, GTV-N dose, and induction chemotherapy were found to be independent prognostic factors for OS, RRFS, and DMFS, respectively, in the Re-irradiation group.

**Conclusions:**

For NPC patients with isolated regional lymph node recurrence after initial radiotherapy, those who underwent surgery had survival prognosis similar to those who underwent re-radiotherapy with or without chemotherapy. A prospective study is needed to validate these findings.

**Supplementary Information:**

The online version contains supplementary material available at 10.1186/s12885-024-12259-w.

## Introduction

Nasopharyngeal carcinoma (NPC) is one of the most common malignant tumors in the Asian population, particularly in southern China and Southeast Asia [1]. Given its high sensitivity to ionizing radiation, radiotherapy is the primary treatment [2]. Owing to the rich lymphatic drainage in the nasopharynx, 49–85% of patients exhibit cervical lymph node metastasis at the initial diagnosis [3]. Despite receiving curative treatment primarily based on radiotherapy, 5–18% of patients still experience regional lymph node recurrence [4, 5].

Several studies have suggested that for suitable patients, surgical resection is the preferred treatment in case of isolated regional lymph node recurrence nasopharyngeal carcinoma (irrNPC). The most common surgical approach is radical neck dissection [6]. Postoperatively, the decision for observation, adjuvant chemotherapy, or radiotherapy is made based on the presence of adverse prognostic factors. For unresectable patients, re-irradiation with or without chemotherapy is the mainstay treatment [6, 7]. However, clinical studies comparing the optimal treatment for irrNPC after radiotherapy are lacking.

To comprehensively assess the therapeutic efficacy of initial radiotherapy in irrNPC patients, we conducted a retrospective study of 124 patients who underwent surgery or re-irradiation, aiming to analyse the clinical characteristics and prognostic factors that can predict survival in irrNPC patients.

## Materials and methods

### Patient characteristics

In this study, data were retrospectively collected from patients with isolated regional lymph node recurrent nasopharyngeal carcinoma who were diagnosed and treated at Fujian Cancer Hospital between January 2010 and December 2020. The inclusion criteria were as follows: 1) had regional lymph node recurrence confirmed by pathology for at least 6 months after initial curative radiotherapy; 2) lacked local nasopharyngeal recurrence and/or distant metastasis; 3) had complete imaging data available at the time of initial treatment and recurrence, including magnetic resonance imaging (MRI), computed tomography (CT) and/or positron emission tomography-CT (PET-CT); and 4) had received treatment primarily consisting of surgery or re-irradiation after recurrence. Patients were categorized into Surgery and Re-irradiation groups based on the treatment modality received after recurrence. Recurrence staging was determined according to the 8th edition of the American Joint Committee on Cancer (AJCC) TNM staging system.

Regional lymph node recurrence was defined as the complete regression of neck lymph nodes after curative radiotherapy, followed by the reappearance of the original neck lymph nodes or the appearance of new lymph nodes at least 6 months later, as confirmed by fine-needle aspiration or tissue biopsy [8]. The radiation dose required to reach the recurrence gross tumor volume (rGTV) during re-irradiation was calculated, and the dose-volume histogram (DVH) was analyzed. Failure patterns were defined as "in-field" if at least 95% of the rGTV was located inside the 95% isodose curve of the prior radiotherapy, “marginal” if 20%-95% of the rGTV was within the 95% isodose, or “out-field” if less than 20% of the rGTV was within the 95% isodose [8, 9]. The diagnostic criterion for radiologic extra-nodal extension (rENE) was based primarily on the standards set in our previous research [10]. In brief, rENE refers to the presence of a matted lymph node and infiltration into adjacent structures, and the results were analyzed by at least two experienced radiologists. This retrospective study was approved by the Ethics Committee of Fujian Cancer Hospital, and written informed consent was obtained from all patients (approval no: K2023-007–01).

### Treatment modalities

The primary treatment modalities for irrNPC patients include surgery and re-irradiation, either alone or in combination [7]. Surgical approaches include radical neck dissection (RND), modified radical neck dissection (MRND), selective neck dissection (SND), and lymph node resection (LNR) [11]. The specific criteria for determining whether irrNPC patients were suitable for surgical resection included the following aspects: 1) All patients were to undergo multidisciplinary consultation to assess whether the recurrent lymph node lesions could be completely excised, and to evaluate the accessibility and surgical difficulty. 2) The patient's overall condition was evaluated to determine the feasibility and safety of surgery. 3) Additionally, patient preferences were to be taken into account when considering treatment options. Postoperative radiotherapy and chemotherapy were administered by the attending physician according to the depth of tumor invasion and the extent of tumor dissection. In general, for patients with positive surgical margins or a greater risk of recurrence, such as those with larger recurrent tumor volumes or radiologic extra-nodal extension, the attending physician could opt for postoperative re-irradiation or chemotherapy to improve local control. Among patients in the Surgery group, 10 patients (50%) received docetaxel/paclitaxel and platinum (TP) chemotherapy regimen, 6 patients (30%) received gemcitabine and platinum (GP) regimen, 3 patients (15%) received an S-1 regimen, and only 1 patient (5%) received a platinum and 5-Fu (PF) regimen.

The gross tumor volume in the neck (GTV-N) was defined as the lesion volume in the regional lymph nodes identified through CT, MRI, or PET-CT. The clinical target volume in the neck (CTV-N) was defined as the region containing the draining lymph nodes, and there was no standardized definition for CTV in our institution's treatment protocol. Generally, only the lymph drainage area of the GTV-N was irradiated for prevention according to the choice of the attending physician [12]. The planning target volume (PTV) was defined as the planning target volume, including setup error and physiological activity, and the margin of the PTV was 3 mm [13]. The prescribed dose range was 60–70 Gy for GTV-N and 50–54 Gy for CTV-N, with 30–35 fractions. Similarly, the decision to administer chemotherapy was left to the discretion of the attending physician. For concurrent chemoradiotherapy (CCRT), the most commonly used regimen was tri-weekly platinum-based treatment (cisplatin 80 mg/m^2^ for 3 days or nedaplatin 80–100 mg/m^2^) [14]. The regimens for induction chemotherapy (IC) included GP, docetaxel /paclitaxel, platinum and 5-Fu (TPF), PF, and TP. Subsequent CCRT or radiotherapy alone was performed depending on the patient's response to induction chemotherapy and treatment tolerance to CCRT.

### Follow-up

Overall survival (OS) was defined as the interval from the diagnosis date of first regional lymph node recurrence to the date of death for any reason or the last follow-up; regional recurrence-free survival (RRFS) was defined as the time from the diagnosis date of regional lymph node recurrence to the date of the next occurrence of regional lymph node recurrence or the last follow-up; and distant metastasis-free survival (DMFS) was defined as the time from the diagnosis date of regional lymph node recurrence to the date of distant metastasis or the last follow-up.

Follow-up was performed every 3 months for the first 2 years after the end of recurrence treatment, every 6 months during years 3 through 5, and annually thereafter. The follow-up included physical examination, nasopharyngoscopy, MRI or CT of the nasopharynx and neck, CT of the lung, and abdominal color Doppler ultrasound, as well as examination of the patient's medical records to determine the patient's condition.

### Statistical analysis

The data analysis was performed using SPSS 26.0 software (SPSS, Inc., Chicago, IL, USA) and R software 4.2.1. Group differences were assessed using the chi-square test. Survival curves were generated using the Kaplan‒Meier method, and differences in survival were evaluated using the log-rank test. Covariates that were significantly associated (*P* < 0.05) with prognosis were initially identified through univariate analysis and subsequently included in the Cox proportional hazards model to assess the effect of prognostic factors. The cut-off value of plasma EBV-DNA was defined by receiver operating characteristic (ROC) curves with Youden’s index using MedCalc software, version 20.123 (https://www.medcalc.org/). All tests were two-sided, and a *P* value less than 0.05 was considered statistically significant.

## Results

### Genera information

**Fig. 1:**
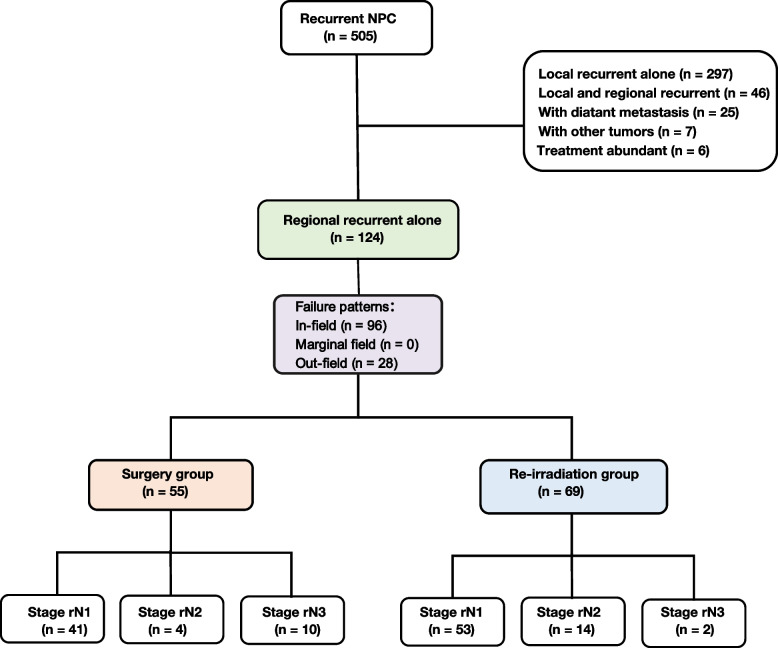
Flow of the study selection process

A total of 124 irrNPC patients were enrolled in this study (Fig. 1). The median time interval between the initial treatment and regional lymph node recurrence was 31 months (ranging from 8 to 278 months). There were 96 patients (77.4%) who experienced in-field recurrence and 28 patients (22.6%) who experienced out-field recurrence (9 in the Surgery group and 19 in the Re-irradiation group). Among the patients with out-field recurrence, 17 had level VIII recurrence, 7 had level I recurrence, and 4 had both level VIII and I recurrence. A total of 12 patients in the cohort had retropharyngeal lymph node recurrence among those with in-field recurrence. In the entire cohort, 55 patients (44.4%) underwent surgery after recurrence (Surgery group), while 69 patients (55.6%) received re-irradiation (Re-irradiation group). Of note, in the Re-irradiation group, 2 surgically resectable patients opted for radiotherapy for personal reasons.

**Fig. 2:**
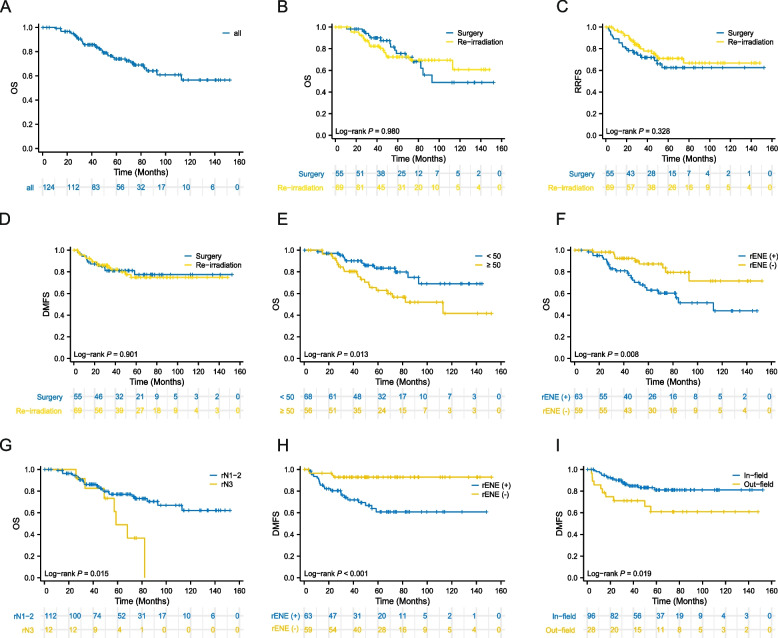
**A** Kaplan‒Meier curves of overall survival (OS) in this cohort. **B-D** Comparisons of OS, regional recurrence-free survival (RRFS) and distant metastasis-free survival (DMFS) between the Surgery group and Re-irradiation group of patients with isolated regional lymph node recurrent nasopharyngeal carcinoma (irrNPC). **E-I** K‒M curves of OS and DMFS in patients with irrNPC stratified by age at recurrence, rENE, rN classification and failure patterns

The majority of recurrent lymph node (rN) classifications were N1 (75.8%), whereas the rN3 classification was more common in the Surgery group and more patients who received combined chemotherapy after recurrent were in the Re-irradiation group (Table 1). The proportion of patients with rENE in the Re-irradiation group was slightly higher than that in the Surgery group (54.4% vs. 48.1%). The analysis of clinical baseline characteristics revealed no significant differences in gender, age, initial T stage, N stage, clinical stage, rENE, failure patterns, or pretreatment Epstein–Barr virus (EBV) DNA between the two groups (*P* > 0.05; Table 1). The median follow-up time was 70.3 months, the mean survival time was 60.8 ± 3.2 months, and the 5-year OS, RRFS, and DMFS rates were 74.0%, 67.4%, and 76.0%, respectively (Fig. 2A). There was no significant difference in 5-year OS (75.6% vs. 72.4%,* P* = 0.973), RRFS (62.7% vs. 71.1%, *P* = 0.330), or DMFS (77.4% vs. 74.9%, *P* = 0.901) between the Surgery group and the Re-irradiation group (Fig. 2B-D).

**Table 1:** Clinicopathological parameters of 124 NPC patients with regional failure

Parameters	Subgroup	Surgery group	Re-irradiation group	*P*
		**NO. (%)**	**NO. (%)**	
Age at recurrence (y)	< 50	32 (58.2)	36 (52.2)	0.587
	≥ 50	23 (41.8)	33 (47.8)	
Gender	Male	45 (81.8)	48 (69.6)	0.146
	Female	10 (18.2)	21 (30.4)	
Initial T classification	T1	12 (23.5)	8 (12.3)	0.433
	T2	20 (39.2)	26 (40.0)	
	T3	14 (27.5)	22 (33.8)	
	T4	5 (9.8)	9 (13.8)	
Initial N classification	N1	9 (18.0)	13 (20.3)	0.603
	N2	23 (46.0)	23 (35.9)	
	N3	18 (36.0)	28 (43.8)	
Initial Clinical stage	I	1 (2.0)	0 (0)	0.661
	II	4 (8.0)	5 (7.7)	
	III	23 (46.0)	26 (40.0)	
	IV	22 (44.0)	34 (53.3)	
rN classification	rN1	41 (75.4)	53 (76.8)	0.003
	rN2	4 (7.3)	14 (20.3)	
	rN3	10 (18.2)	2 (2.9)	
Chemotherapy before recurrent	No	6 (11.3)	5(7.5)	0.534
	Yes	47 (88.7)	62 (92.5)	
rENE	Without	28 (51.9)	31 (45.6)	0.467
	With	26 (48.1)	37 (54.4)	
Failure patterns	In-field	46 (83.6)	50 (72.5)	0.194
	Out-field	9 (16.4)	19 (27.5)	
Chemotherapy at recurrent	No	35 (63.6)	8 (11.6)	< 0.001
	Yes	20 (36.4)	61 (88.4)	
Pretreatment EBV-DNA	< 589	17 (50.0)	31 (58.5)	0.510
	≥ 589	17 (50.0)	22 (41.5)	

*Abbreviation*: *rENE* radiologic extra-nodal extension

### Survival analysis of the entire population

Univariate analysis revealed that age at recurrence ≥ 50 years, rN3 classification, and the presence of rENE were significant adverse prognostic factors for OS (Table 2, Fig. 2E-G). Pretreatment EBV-DNA was an independent prognostic factor for RRFS. The statistically significant adverse prognostic factors for DMFS were the presence of rENE and out-field recurrence (F ig. 2H-I).

**Table 2:** Univariate analysis of potential prognostic factors

Characteristics	Subgroups	OS		RRFS		DMFS	
		**5-year (%)**	***P***	**5-year (%)**	***P***	**5-year (%)**	***P***
Gender	Male	72.5	0.380	67.1	0.810	74.1	0.231
	Female	78.3		68.2		82.7	
Age at recurrence (y)	< 50	83.4	0.013	70.3	0.497	74.2	0.677
	≥ 50	62.9		63.6		78.7	
Treatment after recurrent	Surgery	75.6	0.973	62.7	0.330	77.4	0.901
	Re-irradiation	72.4		71.1		74.9	
Chemotherapy before recurrent	No	78.8	0.830	63.6	0.389	79.5	0.720
	Yes	73.5		68.0		74.5	
rN classification	rN1-2	77.1	0.015	66.4	0.301	76.3	0.668
	rN3	48.9		76.2		74.1	
rENE	Without	87.3	0.007	75.9	0.364	92.9	0.001
	With	63.1		60.9		60.8	
MAD of recurrent lymph nodes	< 3 cm	74.9	0.487	72.5	0.214	74.7	0.861
	≥ 3 cm	73.0		61.9		803	
Failure patterns	In-field	74.1	0.732	70.9	0.168	81.0	0.019
	Out-field	72.9		56.2		60.9	
Bilateral of LN	No	73.9	0.515	68.0	0.618	75.7	0.632
	Yes	74.7		64.2		78.9	
Chemotherapy after recurrent	No	73.2	0.725	63.6	0.960	75.6	0.747
	Yes	73.7		68.6		75.1	
Pretreatment EBV-DNA	< 589	82.1	0.158	65.0	0.045	76.9	0.795
	≥ 589	70.9		89.1		84.2	

*Abbreviation*: *OS* overall survival, *RRFS* regional recurrence-free survival, *DMFS* distant metastasis-free survival, *rENE* radiology extra-nodal extension, *CR* complete response, *PR* partial response, *SD* stable disease, *PD* progression of disease, *LN* lymph node

Cox regression showed that the rENE status was an independent prognostic factor for OS (HR 2.64, 95% CI 1.21–5.74; *P* = 0.014) and DMFS (HR 4.87, 95% CI 1.65–14.40; *P* = 0.004) in irrNPC patients (Table 3). Compared with patients without rENE, patients with rENE had shorter 5-year OS (87.3 vs. 63.1) and DMFS (92.9% vs. 60.8%). Additionally, age at recurrence (HR 2.23, 95% CI 1.08–4.59;* P* = 0.030) and rN classification (HR 2.42, 95% CI 1.02–5.76; *P* = 0.045) were also found to be independent prognostic factors for OS. Patients with the rN3 classification had significantly worse 5-year OS than did those with the rN1-2 classification (77.1% vs. 48.9%). However, multifactorial analysis indicated no significant difference in the prognosis for RRFS by pretreatment EBV-DNA levels.

**Table 3:** Multivariate analysis of potential prognostic factors

	**Factors**	**HR (95%CI)**	***P***
**OS**	Age at recurrence (< 50 vs. ≥ 50 years)	2.23 (1.08–4.59)	0.030
	rENE (without vs. with)	2.64 (1.21–5.74)	0.014
	rN classification (rN1–2 vs. rN3)	2.42 (1.02–5.76)	0.045
**RRFS**	Pretreatment EBV-DNA (< 589 vs ≥ 589)	0.341 (0.11–1.03)	0.056
**DMFS**	rENE (without vs. with)	4.87 (1.65–14.40)	0.004
	Failure patterns (in-field vs. out-field)	2.20 (0.97–4.99)	0.060

*Abbreviation*: *HR* hazard ratio, *CI* confidence interval, *OS* overall survival, *RRFS* regionl recurrence-free survival, *DMFS* distant metastasis-free survival, *rENE* radiology extra-nodal extension

### Establishment of a predictive nomogram model

**Fig. 3:**
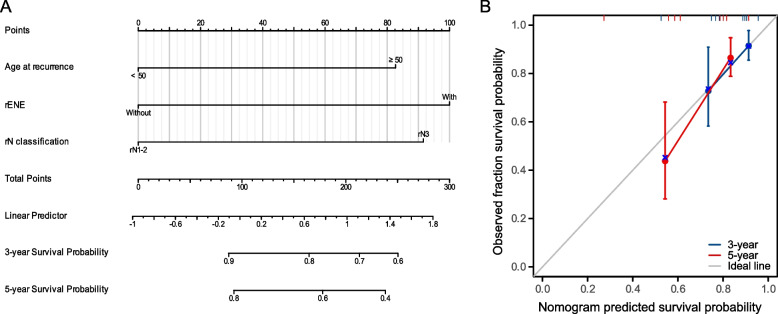
**A** Nomogram model for overall survival based on age at recurrence and the rENE and rN classifications. **B** Calibration curves for predicting overall survival at 3 and 5 years

To enhance prognostication of OS in irrNPC patients, we attempted to establish a nomogram model by integrating prognostic factors, including age at recurrence, rENE and the rN classification (Fig. 3A). A calibration plot was then drawn to estimate the prediction accuracy of this nomogram. The C-index (concordance index) for 3- and 5-year OS was 0.69, suggesting that the nomogram had favorable performance in predicting the survival probability of patients with distant metastasis (Fig. 3B).

### Subgroup analysis in the Surgery group

Among the 55 patients in the Surgery group, 22 (40.0%) patients underwent RND and MRND, and 33 (60.0%) patients underwent SND and LNR. Twenty patients received combined chemotherapy, and 6 patients (3 with RND and 3 with SND) received postoperative re-irradiation at a dose of 50 to 66 Gy (mean 55.4 Gy).

Univariate analysis of various potential influencing factors, including age at recurrence, rN classification, rENE status, recurrence combined with chemotherapy, postoperative re-irradiation, failure patterns, surgical modalities and so on, was performed (Supplementary Table 1). The statistically significant clinical characteristics (*P* < 0.05) were included in the multivariate analysis (Table 4). Notably, RND + MRND vs. SND + LNR had no significant effect on survival, as indicated by the difference in 5-year OS (64.5% vs. 83.4%, *P* = 0.140). Multivariate analysis revealed that the rN classification was an independent prognostic factor for OS (hazard ratio (HR) 3.72, 95% CI 1.24–14.55; *P* = 0.026). Failure pattern (HR 2.86, 95% CI 1.02–8.03; *P* = 0.047) and bilateral lymph node recurrence (HR 6.35, 95% CI 1.65–24.41; *P* = 0.007) were found to be independent influencing factors for RRFS.

**Table 4:** Multivariate analysis of patients in the Surgery group

*Variables*	*HR (95%CI)*	*P*
**Overall survival**		
rN classification (rN1–2 vs. rN3)	3.72 (1.24–14.55)	0.026
rENE (without vs. with)	2.86 (0.75–10.85)	0.056
**Regional relapse-free survival**		
Failure patterns (in-field vs. out-field)	2.86 (1.02–8.03)	**0.047**
Bilateral of LN (no vs. yes)	6.35 (1.65–24.41)	**0.007**
Chemotherapy at recurrence (without vs. with)	2.41 (0.92–6.34)	0.074
**Distant metastasis-free survival**		
rENE (without vs. with)	4.22 (0.88–20.17)	0.072
Number of positive LN (< 6 vs. ≥ 6)	3.26 (0.91–11.72)	0.070

*Abbreviation*: *HR* hazard ratio, *CI* confidence interval, *LN* lymph node, *rENE* radiology extra-nodal extension

### Subgroup analysis of the Re-irradiation group

In the Re-irradiation group, 64 patients received intensity-modulated radiation therapy (IMRT), 4 patients received conventional radiotherapy, and 1 patient with right retropharyngeal lymph node recurrence received ultrasound-guided high-dose brachytherapy (30 Gy with 1 fraction) after multidisciplinary team discussion and according to the patient's wishes. This patient experienced right retropharyngeal lymph node recurrence three years post-treatment. Five patients received re-irradiation with a GTV-N less than 60 Gy due to intolerance. Sixty-one patients (88.4%) also received chemotherapy, 47 of whom received induction chemotherapy, 45 of whom received concurrent chemotherapy, and 8 of whom received adjuvant chemotherapy.

The clinical factors that were significant in univariate analysis, including age at recurrence, combination of chemotherapy during radiotherapy, maximal axial diameter (MAD) of recurrent lymph nodes, failure pattern, rENE status and GTV-N dose, etc., were included in multivariate analysis (Supplementary Table 2). The results showed that age at recurrence (HR 3.35, 95% CI 1.19–9.42; *P* = 0.022) was significantly associated with OS. A GTV-N ≥ 60 Gy was significantly associated with increased RRFS (hazard ratio (HR) 0.18, 95% CI 0.05–0.68;* P* = 0.011). In addition, patients who relapsed and received induction chemotherapy (HR 0.26, 95% CI 0.09–0.75; *P* = 0.013) had longer DMFS (Table 5). Notably, there was no significant difference in survival between the in-field and out-field failure patterns in the Re-irradiation group.

**Table 5:** Multivariate analysis of patients in the Re-irradiation group

Variables	HR (95%CI)	*P*
**Overall survival**		
Age at recurrent (< 50 vs. ≥ 50 years)	3.35 (1.19–9.42)	**0.022**
**Regional relapse-free survival**		
GTV-N dose (< 60 vs. ≥ 60 Gy)	0.18 (0.05–0.68)	0.011
**Distant metastasis-free survival**		
Induction chemotherapy at recurrent (without vs. with)	0.26 (0.09–0.75)	0.013

*Abbreviation*: *HR* hazard ratio, *CI* confidence interval

## Discussion

Numerous studies have explored the optimal treatment for locally recurrent nasopharyngeal carcinoma [15, 16]. However, to our knowledge, this study represents the first to compare the long-term efficacy of surgery versus re-irradiation in simple irrNPC patients after initial radiotherapy. Our results showed that irrNPC patients achieved similar long-term disease control and survival outcomes, including OS, RRFS, and DMFS, after aggressive treatment with surgery or re-irradiation. Age at recurrence, rENE, and rN classification were the most important prognostic factors for irrNPC patients. Additionally, the nomogram based on these factors performed well in predicting the prognosis of patients with irrNPC.

Emerging studies have shown that radiological extracapsular extension (rENE), similar to pathological extracapsular extension (pENE), is an independent adverse prognostic factor for NPC patients [14, 17–19]. In this study, 63 (50.8%) irrNPC patients had rENE, and we found that rENE was an important adverse factor for OS and DMFS. The 5-year differences in OS and DMFS based on the presence of rENE were 24.6% and 32.1%, respectively. For irrNPC patients with rENE, it is unclear whether surgical treatment or re-irradiation is better. Besides, whether it is necessary to combine chemotherapy, targeted therapy, or immunotherapy based on surgery or re-radiotherapy to improve the tumor control rate still needs to be further studied through prospective, multicenter, large sample clinical trials [17]. Our study is the first research to explore the impact of rENE on irrNPC, allowing for earlier prognostic assessment before surgery or re-irradiation.

Research studies have reported 5-year OS rates for patients with irrNPC ranging from 58% to 87.1% [20–23], which is in line with our study. According to the Chinese Society of Clinical Oncology (CSCO) guidelines, neck lymph node dissection is a critical curative treatment for irrNPC, and postoperative radiotherapy after lymph node dissection is also a viable treatment option [7]. Our study demonstrated that the efficacy of neck lymph node dissection with or without postoperative radiotherapy was similar to that of re-irradiation alone. Furthermore, there was no significant difference in whether postoperative re-irradiation was administered after neck lymph node dissection, aligning with previous findings [24]. In clinical practice, most cases of regionally recurrent disease are technically resectable [25]. Radical neck dissection (RND) or modified radical neck dissection (MRND) are widely considered to be the mainstays of treatment [6, 26]. Selective neck dissection (SND) has become increasingly popular in recent years [11]. In our study, different surgical modalities did not provide any “protective” effect on the survival prognosis of irrNPC patients. Among patients in the Surgery group, the rN classification was the only factor significantly associated with OS, with a marked decrease in survival rates for patients in the rN3 classification. In addition, patients with out-field recurrence and bilateral lymph node recurrence had significantly worse regional control rates. Therefore, for patients with a high recurrent staging, further research is needed to determine whether adjuvant chemotherapy, radiotherapy, or immunotherapy can prolong survival.

Re-irradiation is one of the main treatment modalities for irrNPC patients, especially for those patients with a recurrence interval of more than 1 year [7, 27]. Sham et al. reported that re-irradiation for regional lymph node recurrence yielded poor results, with a 5-year OS of only 19.7%. For patients with lymph nodes larger than 4 cm^2^, the 5-year RRFS was as low as 16% [28]. Similarly, Daniel et al. reported that the 3-year RRFS after re-irradiation for irrNPC following RND was 24%, whereas it was 65% for patients receiving RND alone [29]. However, these studies employed conventional radiotherapy techniques, and the median dose to the recurrent neck lymph nodes ranged from 51 to 53.4 Gy, which was very different from the radical dose of intensity-modulated radiation therapy (IMRT) used in most patients in our study. The conclusion drawn in the study by Xiao et al. [5] is consistent with our findings that patients who underwent surgery had similar OS to those who received re-irradiation with IMRT. Notably, only a small percentage (8.6%) of patients in their study received re-irradiation, which could introduce significant bias. In our study, the use of induction chemotherapy before re-irradiation reduced the risk of distant metastasis in the re-irradiation group. Thus, for patients with a high risk of metastasis, such as those with a high risk of metastasis according to rN3 classification or the presence of rENE, induction chemotherapy may be advised before re-irradiation.

This study has several limitations. First, as a retrospective study, it is susceptible to selection bias. The absence of a detailed analysis of treatment-related toxicity in patients undergoing re-irradiation for isolated regional recurrence is another limitation. Moreover, this was a single-center study conducted in a high-incidence region, possibility limiting the generalizability of the results to a broader population. These findings are more likely to apply to patients with WHO type III nasopharyngeal carcinoma, which is more common in southern China. Additional studies from different centers or well-designed prospective studies are needed to validate these results.

## Conclusion

In conclusion, our study suggested that, compared with patients treated with surgery, irrNPC patients treated with aggressive radiotherapy alone or in combination with chemotherapy can achieve similar outcomes. These findings warrant further validation through multicenter, large-sample studies.

### Supplementary Information

**Supplementary Material 1.**

**Supplementary Material 2.**

## Data Availability

The raw data supporting the conclusions of this article will be made available by the authors. Further inquiries can be directed to the corresponding authors.

## Abbreviations

irrNPC: Isolated regional lymph node recurrent nasopharyngeal carcinoma
OS: Overall survival
RRFS: Regional recurrence-free survival
DMFS: Distant metastasis-free survival
ROC: Receiver operating characteristic
rENE: Radiologic extra-nodal extension
pENE: Pathological extracapsular extension
rGTV: Recurrence gross tumor volume
rN: Recurrent lymph node
DVH: Dose-volume histogram
RND: Radical neck dissection
MRND: Modified radical neck dissection
SND: Selective neck dissection
LNR: Lymph node resection
MRI: Magnetic resonance imaging
CT: Computed tomography
PET-CT: Positron emission tomography-CT
GTV: Gross tumor volume
CTV: Clinical target volume
PTV: Planning target volume
IMRT: Intensity-modulated radiation therapy
IC: Induction chemotherapy
CCRT: Concurrent chemoradiation
MAD: Maximal axial diameter
EBV: Epstein–Barr virus
TP: Docetaxel/paclitaxel and platinum
GP: Gemcitabine and platinum
TPF: Docetaxel/paclitaxel, platinum and 5-Fu
PF: Platinum and 5-Fu
CR: Complete response
PR: Partial response
SD: Stable disease
PD: Progressive disease
LN: Lymph node
