# Supplementary material for: Efficacy of salvage surgery versus re-irradiation for isolated regional lymph node recurrence in patients with nasopharyngeal carcinoma
Source: BMC Cancer. 2024 Apr 16;24:483. doi: 10.1186/s12885-024-12259-w (PMC11022380; doi:10.1186/s12885-024-12259-w)
Supplement: Supplementary file 1 — Supplementary Material 1. [file 12885_2024_12259_MOESM1_ESM.docx]

| **Table S1** Univariate analysis of potential prognostic factors for patients in Surgery group | | | | | | |
| --- | --- | --- | --- | --- | --- | --- |
| **Variables** | **OS** | | **RRFS** | | **DMFS** | |
|  | **HR (95%CI）** | ***P*** | **HR (95%CI）** | ***P*** | **HR (95%CI）** | ***P*** |
| Gender (male vs. female) | 0.18 (0.02-1.40) | 0.101 | 0.47 (0.11-2.05) | 0.313 | 0.03 (0-15.90) | 0.279 |
| Age at recurrence (< 50 vs. ≥ 50 years) | 1.54 (0.60-4.27) | 0.402 | 1.49 (0.59-3.76) | 0.399 | 0.52 (.34-1.95) | 0.330 |
| rN classification (rN1–2 vs. rN3) | 4.04 (1.34-12.15) | 0.013 | 0.47 (0.11-2.03) | 0.311 | 1.83 (0.48-6.89) | 0.373 |
| rENE (without vs. with) | 4.29 (1.19-15.46) | 0.026 | 2.25 (0.83-6.10) | 0.110 | 5.51 (1.19-25.56) | 0.029 |
| Chemotherapy at recurrent (without vs. with) | 1.01 (0.36-2.85) | 0.988 | 2.68 (1.06-6.80) | 0.038 | 2.11 (0.64-6.92) | 0.218 |
| Postoperative re-irradiation (without vs. with) | 0.41 (0.05-3.11) | 0.385 | 0.36 (0.04-2.68) | 0.315 | 0.70 (0.09-5.48) | 0.734 |
| MAD of recurrent lymph nodes (< 3 vs. ≥ 3cm） | 1.20 (0.41-3.44) | 0.749 | 2.53 (0.94-6.80) | 0.066 | 1.34 (0.41-4.38) | 0.632 |
| Failure patterns (in-field vs. out-field) | 2.07 (.56-7.69) | 0.278 | 3.48 (1.29-9.42) | 0.004 | 2.21 (0.58-8.36) | 0.243 |
| Bilateral of LN (no vs. yes) | 0.04 (0-65.57) | 0.393 | 6.29 (1.78-22.29) | 0.003 | 2.92 (0.63-13.53) | 0.172 |
| Surgical modalities (RND+MRND vs. SND+LNR) | 0.46 (0.17-1.29) | 0.140 | 0.58 (0.23-.45) | 0.242 | 0.34 (0.01-.15) | 0.083 |
| Number of positive LN (< 6 vs. ≥ 6) | 2.09 (0.58-7.51) | 0.258 | 2.56 (0.82-7.99) | 0.105 | 4.18 (1.18-14.88) | 0.027 |
| Pretreatment EBV-DNA (< 589 VS. ≥ 589) | 1.33 (0.24-7.34) | 0.743 | 0.76 (0.17-3.41) | 0.722 | 0.62 (0.10-3.70) | 0.597 |
| Abbreviation: HR hazard ratio; CI confidence interval; OS overall survival; RRFS regionl recurrence-free survival; DMFS distant metastasis-free survival; LN lymph node; rENE radiology extra-nodal extension; MAD maximal axial diameter. | | | | | | |
