# Supplementary material for: Efficacy of salvage surgery versus re-irradiation for isolated regional lymph node recurrence in patients with nasopharyngeal carcinoma
Source: BMC Cancer. 2024 Apr 16;24:483. doi: 10.1186/s12885-024-12259-w (PMC11022380; doi:10.1186/s12885-024-12259-w)
Supplement: Supplementary file 2 — Supplementary Material 2. [file 12885_2024_12259_MOESM2_ESM.docx]

| **Table S2** Univariate analysis of potential prognostic factors for patients in Re-irradiation group | | | | | | |
| --- | --- | --- | --- | --- | --- | --- |
| **Variables** | **OS** | | **RRFS** | | **DMFS** | |
|  | **HR (95%CI）** | ***P*** | **HR (95%CI）** | ***P*** | **HR (95%CI）** | ***P*** |
| Gender (male vs. female) | 1.14 (0.43-3.03) | 0.799 | 1.48 (0.56-3.88) | 0.429 | 0.63 (0.18-2.25) | 0.475 |
| Age at recurrent (< 50 vs. ≥ 50 years) | 3.35 (1.19-9.42) | 0.022 | 1.09 (0.42-2.82) | 0.864 | 1.59 (0.55-4.58) | 0.394 |
| rN classification (rN1–2 vs. rN3) | 1.61 (0.21-12.18) | 0.647 | 0.05 (0-3072.66) | 0.588 | 0.05 (0-18686.82) | 0.642 |
| rENE (without vs. with) | 1.96 (0.74-5.24) | 0.178 | 0.87 (0.33-2.33) | 0.788 | 3.26 (0.90-11.88) | 0.073 |
| Induction chemotherapy at recurrent (without vs. with) | 0.57 (0.22-1.48) | 0.250 | 0.41 (0.15-1.11) | 0.078 | 0.26 (0.09-0.75) | 0.013 |
| Concurrent chemotherapy at recurrent (without vs. with) | 0.98 (0.36-2.64) | 0.965 | 0.50 (0.19-1.34) | 0.167 | 0.83 (0.28-2.48) | 0.740 |
| Adjuvant chemotherapy at recurrent (without vs. with) | 0.22 (0.03-1.69) | 0.147 | 0.56 (0.13-2.47) | 0.444 | 0.31 (0.04-2.35) | 0.254 |
| GTV-N dose (< 60 vs. ≥ 60Gy) | 3.02 (1.07-8.51) | 0.581 | 0.18 (0.05-0.68) | 0.011 | 0.65 (0.08-4.64) | 0.616 |
| MAD of recurrent lymph nodes (< 3cm vs. ≥ 3cm) | 1.21 (0.39-3.72) | 0.744 | 0.83 (0.24-2.92) | 0.770 | 0.59 (0.13-2.65) | 0.486 |
| Failure patterns (in-field vs. out-field) | 1.04 (0.39-2.81) | 0.931 | 0.97 (0.34-2.77) | 0.958 | 1.93 (0.67-5.57) | 0.228 |
| Bilateral of LN (no vs. yes) | 1.01 (0.33-3.07) | 0.988 | 0.71 (0.21-2.49) | 0.595 | 0.26 (0.03-1.99) | 0.195 |
| Grade ≥ 3 acute toxicities (without vs. with) | 0.64 (0.15-2.78) | 0.548 | 1.00 (0.29-3.47) | 0.994 | 1.33 (0.37-4.80) | 0.660 |
| Pretreatment EBV-DNA (< 589 VS. ≥ 589) | 2.07 (0.66-6.61) | 0.211 | 0.15 (0.02-1.20) | 0.074 | 1.13 (0.32-4.02) | 0.850 |
| Abbreviation: HR hazard ratio; CI confidence interval; OS overall survival; RRFS regionl recurrence-free survival; DMFS distant metastasis-free survival; LN lymph node; rENE radiology extra-nodal extension; MAD maximal axial diameter. | | | | | | |
